# Development and validation of the Trust in Government measure (TGM)

**DOI:** 10.1186/s12889-023-16974-0

**Published:** 2023-10-17

**Authors:** Kathleen E. Burns, Patrick Brown, Michael Calnan, Paul R. Ward, Jerrica Little, Gustavo S. Betini, Christopher M. Perlman, Helena Godinho Nascimento, Samantha B. Meyer

**Affiliations:** 1 School of Public Health Sciences, University of Waterloo, 200 University Ave West, Waterloo, ON N2L 3G1 Canada; 2 Department of Sociology, University of Amsterdam, Amsterdam, 1012 WX Netherlands; 3 School of Social Policy, Sociology and Social Research, University of Kent, Canterbury, CT2 7NB UK; 4 Research Centre for Public Health, Equity and Human Flourishing, Torrens University, 88 Wakefield St, Adelaide, SA 5000 Australia

**Keywords:** Trust, OECD, Measure, Federal, Government, Validation

## Abstract

**Background:**

Trust in government is associated with health behaviours and is an important consideration in population health interventions. While there is a reported decline in public trust in government across OECD countries, the tools used to measure trust are limited in their use for informing action to (re)build trust, and have limitations related to reliability and validity. To address the limitations of existing measures available to track public trust, the aim of the present work was to develop a new measure of trust in government.

**Methods:**

Fifty-six qualitative interviews (Aug-Oct 2021; oversampling for equity-deserving populations) were conducted to design a national survey, including factor analyses and validation testing (N = 878; June 1-14th 2022) in Canada.

**Results:**

The measure demonstrated strong internal consistency (α = 0.96) and test validity (CFI = 0.96, RMSEA = 0.09, SRMR = 0.03), suggesting that trust in government can be measured as a single underlying construct. It also demonstrated strong criterion validity, as measured by significant (p < 0.0001) associations of scores with vaccine hesitancy, vaccine conspiracy beliefs, COVID-19 conspiracy beliefs, trust in public health messaging about COVID-19, and trust in public health advice about COVID-19. We present the Trust in Government Measure (TGM); a 13-item unidimensional measure of trust in Federal government.

**Conclusions:**

This measure can be used within high-income countries, particularly member countries within the OECD already in support of using tools to collect, publish and compare statistics. Our measure should be used by researchers and policy makers to measure trust in government as a key indicator of societal and public health.

## Background

Trust in government is critical for societal functioning and the health of the population [1]. While trust in healthcare is most often cited as shaping health behaviour [2] trust in government too has been associated with health behaviours and as such, is an important consideration in interventions targeting population health. For example, trust in government was found to be associated with lower COVID-19 infection rates and higher levels of vaccine coverage in middle- and high-income countries with higher vaccine availability [3]. These associations can be understood, in turn, through the association of trust in government with increased social cohesion and interpersonal trust between citizens [4, 5], and with public acceptance of government policy [6, 7] and recommended health behaviours [8–15]. Trust in government is also positively associated with trust in public health messages and trust in health advice from the government [9], and negatively associated with believing misinformation and conspiracy theories, and accessing resources that are deemed to be inaccurate [16]. Trust, therefore, may act as a barometer for public support of government initiatives that shape health at a population level.

Trust in government has reportedly declined globally over time. In 2020, the Organisation for Economic Co-Operation and Development (OECD) found that only 51% of citizens in OECD countries trusted their national government; estimated to be 60% in Canada [7]. Within Canada, the location of the present study, trust in government is the most volatile in terms of institutional trust categories, compared to businesses, non-government organizations (NGOs), and the media [17]. The decline in governmental trust may be attributed specifically to the decline in trust in political leadership in Canada (from 39% to 2020 to 33% in 2022 [18, 19]). Within Canada, the decline in public trust is likely influenced by increasing polarization in public perceptions as they relate to the government’s response to COVID-19, as reports indicated public concern regarding the effectiveness of government responses to pandemic management [20]. Declines in trust may also be in response to perceptions of inadequate government attention to pressing social issues in Canada; for example, climate change and Reconciliation with Indigenous Peoples for historical injustices (e.g., the discovery over unmarked graves of Indigenous children found at the sites of former Government residential schools in Canada).

Despite the importance of governmental trust for societal functioning and health behaviour, existing tools are limited in their use for informing action to (re)build trust. Current measurement of trust in government as an institution in empirical literature largely relies on a single item that is dichotomized [21] or on a scale [22–24], limiting our understanding of *why* people trust. Other measures look at combined averages of reported trust in individuals *and* institutions (e.g., politicians, current government, and civil servants [25, 26]), trust as it relates to specific government actions [27], or they measure the similar but semantically distinct concepts ‘mistrust’ and ‘distrust’ [26, 28]. Existing measures used for research are also largely adapted from the World Values Survey [23, 29, 30] and the OECD TrustLab [31]; the former measures confidence in institutions over trust, while the latter uses a single item ‘how much trust do you have in the government?’. Further, many measures of trust in government do not report on the reliability or validity of measures, or in other cases, the theoretical and/or conceptual frameworks used to inform these measures [32, 33]. Finally, many of the existing trust measures look solely at interpersonal trust and are not used to measure trust in larger institutions or in governmental institutions comprising many actors and agencies [34–37].

Government interventions to improve trust need to be rooted in data collected using valid measures that account for dimensions of trust, rather than single items, so that we can identify items within these measures that shift in response to government action and/or negatively impact trust. The aim of the present work was to address current limitations and develop the Trust in Government Measure (TGM). While recent measures (e.g., [38]) overcome some of the noted limitations, we collect data generated post-COVID and following major social movements (e.g., in response to the murder of George Floyd) that changed discussions regarding trust in social institutions. Relatedly, we also oversample sub-populations historically disadvantaged by social institutions that might provide more insight into the concept of trust. Our work thus extends existing measures for use with diverse populations to track trust over time. Our goal is to provide a measure that may be used by government to assess levels of trust now and moving forward, and to work towards greater support of government initiatives aimed at promoting the health of the population.

The remainder of this article is structured to present two studies; one is the development of the scale (the ‘development study’), and the other is the validation (the ‘validation study’). We present the methods and results for each study and then the final measure - Trust in Government Measure - with a discussion section speaking to the combined contribution of both studies.

### Development of the trust in government measure

#### Conceptual model

Trust occurs at both institutional and interpersonal levels [39] that are most often inextricably linked; that is, trust in individuals (e.g., political leaders), who serve as the face of institutions, to some extent impacts trust in the organization with which they represent, though trust in whom or what comes first remains disputed [39, 40]. With this in mind, however, institutional trust, the focus of the present research, is argued to be based on an individual’s perceptions of an organization [38, 41], going beyond whether someone has a positive or negative attitude toward the institution [42] – that is, one might trust an institution even if they disagree with the operation of the institution (e.g., you may trust your government even if you do not agree with a specific policy). Institutional trust has been described as the extent to which individuals perceive institutions as benevolent, competent, reliable, and responsible toward citizens [5, 42]; this definition, focused on both the competence and care of the organization towards citizens, would suggest that institutional trust is a multidimensional construct – that both care and competence factor into the assessment of the trustworthiness of an institution. Additionally, institutions that are perceived as impartial, fair, and efficient are more likely to be trustworthy and therefore trusted by citizens [4].

Previous research suggests that perceptions of the competence and values - that is, the ability of the government to deliver quality public services, respond to the needs of all citizens and to manage all aspects of uncertainty, including social, economic, and political situations - are critical for public trust [31]. Values that encompass norms of integrity, which include low corruption, high accountability, transparency of policy processes, and fair and equitable treatment of all citizens, are identified as the strongest determinants of governmental trust [7, 31]. These are demonstrated by government in the provision or regulation of public services (responsiveness), anticipation of change and protecting citizens (reliability), using power and public resources ethically (integrity), listening, consulting, engaging, and explaining to citizens (openness), and improving living conditions for all (fairness) [7]. As we note below, these values and norms are consistent with the dimensions of trust in two existing international measures of trust - The ‘Citizen Trust in Government Organizations’ scale [38] and a ‘Trust in Public Health Authorities (TiPHA) scale’ [43] – that we used to guide item generation for our measure. The main dimensions of interest that incorporate these drivers include beneficence, competence [38, 43], and integrity [36, 38], which is supported by two systematic reviews examining trust in social institutions and health professionals and measures of trust in organizations [44, 45].

### Methods for development study

#### Item generation

The team reviewed existing measures of trust in government [44] and identified two measures as a starting point for our work in developing and validating a trust in government instrument in the Canadian context; The ‘Citizen Trust in Government Organizations’ scale [38] and the ‘TiPHA’ [43]. These instruments were used as a starting point because of their inclusion of the critical dimensions of competence, beneficence and integrity, recent development, and potential for adaptability to different contexts [38, 43]. Both measures also have demonstrated high reliability and validity [38, 43].

### Data collection

Interviews (N = 56) were conducted between August and December 2021with a diverse sample of Canadians to explore if dimensions of trust used in existing measures - beneficence, competence [38, 43], and integrity [36, 38] - were consistent in the Canadian context. The goal was to modify/remove/create candidate items based on data from interviews that were not yet captured in the existing measures (e.g., did not reflect Canadian values with regards to trust). In addition to the general population (n = 19), we intentionally recruited subgroups historically disadvantaged by social institutions in Canada in order to obtain insight into the perspective of those most likely to have experience of distrust: n = 7 First Nations, Métis and Inuit; n = 5 LGBT2SQ+; n = 8 low income (< $40,000CAD annual household income); n = 7 Black Canadians; n = 10 newcomers (less than 5 years living in Canada). This allowed us to identify relevant items for our measure, and to ensure that we were not missing dimensions beyond existing measures developed in other jurisdictions.

Participants were recruited through Leger, Canada’s largest and most representative research marketing firm, to gain representation from harder-to-reach populations. Leger recruited potential participants and provided contact information to the research team. Interviews were conducted via telephone or a virtual platform (Cisco Webex, Zoom or Microsoft Teams), depending on the preference of the participant. Key questions included: Do you trust the current federal government? What are they doing well? What are they doing poorly? Do your opinions differ with the current government? If so, how? Do your opinions differ with previous administrations? If so, how? How does being a member of [community – e.g., Black Canadian] affect your trust relationship with the government? Is there anything the current government could to do improve your trust?

Given the period of data collection, responses to interview largely reflected perceptions of government considering their response to COVID-19. As such, COVID-19 provided a case from which participants could draw examples. Our approach to analysis was to interrogate responses to identify the dimensions underpinning discussions of trust, related to COVID-19 or not, in discussions of trust in government broadly. However, as our prior research has demonstrated, within disadvantaged or marginalised populations there are unique factors that challenge trust in government given experiences of oppression and discrimination throughout history [46, 47]. Historical and ongoing systemic oppression likely call into question the integrity and intentions of government and public health, irrespective of COVID-19, to an extent that might not occur in consideration of other institutions.

Interviews were recorded and transcribed verbatim by an agency abiding by a confidentiality agreement. We then underwent a process of conceptual coding with the goal of revising candidate items to finalize the survey for the validation study. We mapped the findings from deductive focused coding using dimensions of competence and beneficence from the ‘Citizen Trust in Government Organizations’ scale [38] and TiPHA scale [45] and a third dimension of integrity from the Citizen Trust in Government Organizations’ scale [38]. In brief, analysts inductively and deductively coded data to identify both existing and emerging dimensions of trust. Conceptual categories were grouped to align with original dimensions items which were reviewed, edited, and removed. New items were created based on inductive codes. Any discrepancies between the data and the original measures were documented, leading to a preliminary survey for use in the validation study.

### Results of development study

Participants were representative of most Canadian Provinces, with Ontario having the highest number of participants (45%). The gender distribution of the participants was 64% female, 34% male, and 2% non-binary. Regarding sexual orientation, 82% of the respondents were heterosexual, 14% were LGBTQS2+ (LGBT), and 4% preferred not to respond. Most participants (38%) preferred not to disclose their political affiliation but 25% cited support for Liberal, ~ 16% for Conservative, ~ 11 the New Democratic Party, ~ 9 for the People’s Party of Canada, and ~ 2 for the Green Party. We value and recognize the potential role of intersectionality in some of the respondents’ answers. However, sample sizes in the present work were too low to permit meaningful analyses.

Based on analysis summaries from the 56 participant interviews, data were found to align with the three dimensions of trust in government as outlined by Grimmelikhuijsen & Knies (2017): competence, beneficence, and integrity. These dimensions were largely evaluated by participants through the government’s communication, decision making, transparency, honesty and delivering on promises. Given the timing of the data collection used in this study, many participants discussed these dimensions through their perceptions of the federal and provincial governments’ responses to the COVID-19 pandemic. Participants mostly discussed and evaluated their trust in government based on their perceptions of governmental communication and decision-making.

Competence was discussed and evaluated by participants through their perceptions of governmental communication and decision-making, largely in discussions related to COVID-19 communication. For example, participants expressed that governmental communication was poor, as the messaging lacked sufficient information, clarity, and consistency. For example:

…the communication could be clearer. And not to be rude or whatever but dumb it down a bit so everybody can understand it. Yeah, because I think sometimes maybe the wording isn’t easy enough for some people to get.

There’s been a lot. It’s been really annoying how often they change, and that can make it difficult to understand what, we as citizens are expected to do because they keep changing, and it also makes us doubt the research that’s going into this, because it seems like the research is constantly changing as well.

Participants also perceived the government’s response to the COVID-19 pandemic as generally ineffective, which negatively impacted their trust in government, and that COVID-19 pandemic measures were not effectively implemented, as some countermeasures were perceived as being lifted too quickly. Other measures were perceived as contradictory or confusing.

I think that particularly in Alberta, we have a [sic] extraordinarily ineffective response to the pandemic and it’s somehow gotten worse as it’s gone through the pandemic. And I think that our leaders in my provincial government are even less trustworthy and they’re either going to either be mishandling or actively hampering handling the pandemic.

… after the first wave, they did rush and reopen many unnecessary things which resulted in us being back to another lock down at all. I feel like then all the government could have done a better job.

I do not [trust the government]. I believe that their health care recommendations in particular were ineffective, especially at the beginning of the pandemic, that they kind of provided some minimum level…of addressing the pandemic at different stages, but that they weren’t really, overly effective. And so I don’t believe that…, based on their past history of the communication and the actions that they took, that I can trust them to manage a pandemic.

Participants discussed beneficence through the provision of resources and decision-making related to COVID-19 responses. More specifically, important factors related to the provision of resources included providing financial support to individuals and businesses during the COVID-19 pandemic. For example:

… And also, yeah, those who have struggled because of the Pandemic, they were all supported from some kind of incentive. And that’s something that I appreciated because in many parts of the world, people lose their jobs during the pandemic and they didn’t have any income and they had to struggle a lot because they lost a job and they didn’t have any income. In Canada, all the people, all people were taken care of even during the pandemic for quite a long time….

On the contrary, they also shared concerns, that impacted their trust, related to the perception that government was putting political agendas ahead of public health. They spoke of a government worthy of trust as one that promoted fairness and equity, where decisions are made with the interest of citizens in mind.

…That blatant implication of politics in public health decisions where politics doesn’t really belong has fostered this sense of distrust and frustration….

Regarding integrity, participants associated a lack of transparency and honesty with a lack of trust in government. Specifically, they spoke of government failing to deliver on stated promises as a factor in losing the trust of citizens.

…But, again, there’s been a lot of that [government] trust that’s been lost over the last bit because of this, just lack of transparency, lack of honesty, so a lot of that has been lost. It’s going to be hard for me to gain it back, at least from what I’ve heard from talking to people.

And I still would have been distrusting [of the government], I think, that this particular government is affected or has been ineffective in helping Canadians and keeping the promises that they made during their last election period and the one before and just being mired in, you know, scandals around the government and different processes, individual people, the government. I, I don’t think I had a lot of trust going into it….

Based on the qualitative interview analysis, we retained items from three existing measures covering three dimensions of trust in government: competence, beneficence, and integrity, as outlined in Table 1. One new item, the government making decisions that support citizen autonomy (related to beneficence), was added based on the qualitative data.

**Table 1:** Factors of trust in government measuring the three dimensions

Dimension	Elements required for survey item development/refinement		
	Grimmelikhuijsen and Knies, 2017	Holroyd et al., 2021	World Values Survey
Competence	Capability Effectiveness Judgment Knowledge/expertise Carrying out duties	Effectiveness Knowledge/expertise Ability to protect public	Carrying out duties
Beneficence	Citizen focus Acting in best interest of citizens	Acting in the best interest of citizens Protecting the public	
Integrity	Sincerity/honesty Deliver on commitments		Transparency and openness

The result was a 17-item measure of trust in government. The measure included 7 questions to evaluate government competence based on perceived capability, effectiveness, judgment [38], knowledge/expertise [38, 43], and carrying out duties [38, 48]; 6 items to evaluate beneficence of the government based on citizen focus [38], acting in the best interest of citizens [38, 43], and protecting the public [43]; and 4 items to evaluate the perceived integrity of the government based on sincerity/honesty, delivering on promises [38], and transparency and openness [48]. The measure of trust in government used for the validation study is provided in Table 2.

**Table 2:** Measure citizen trust in the federal government for validation study

Existing Items	New items
[The municipality of XXX] is capable. Grimmelikhuijsen and Knies, 2017	Q1. The federal government can help citizens in need. Q2. The federal government can protect the health of the population.
[The municipality of XXX] is effective. Grimmelikhuijsen and Knies, 2017	Q3. The federal government communicates with citizens effectively. Q4. The federal government makes decisions that help citizens.
[The municipality of XXX] is skillful. Grimmelikhuijsen and Knies, 2017 People in the government often show poor judgement. World Values Survey, 2017–2021	Q5. The federal government shows good judgment.
[The municipality of XXX] is expert. Grimmelikhuijsen and Knies, 2017	Q6. The federal government has sufficient expertise to lead the country
[The municipality of XXX] carries out its duty very well. Grimmelikhuijsen and Knies, 2017 <Parliament > usually carries out its duties poorly. World Values Survey, 2017–2021	Q7. The federal government carries out its duties very well
[The municipality of XX] acts in the interest of citizens. Grimmelikhuijsen and Knies, 2017	Q8. The federal government acts in the best interest of citizens.
If citizens need help, [the municipality of XX] will do its best to help them. Grimmelikhuijsen and Knies, 2017	Q9. If citizens need help, the federal government will do its best to help them.
N/A-Supporting Autonomy. Emerged from qualitative data.	Q10. The federal government makes decisions that support citizen autonomy (independence).
They do everything they should to protect the health of the population. Holroyd et al., 2021	Q11. The federal government does everything they should to protect the population.
[The municipality of XX] is genuinely interested in the well- being of citizens. Grimmelikhuijsen and Knies, 2017	Q12. The federal government is genuinely interested in the wellbeing of its citizens. Q13. The federal government puts their political agenda ahead of the wellbeing of the population.
[The municipality of XX] approaches citizens in a sincere way. Grimmelikhuijsen and Knies, 2017	Q14. The federal government is truthful in communication with citizens.
[The municipality of XX] keeps its commitments. Grimmelikhuijsen and Knies, 2017	Q15. The federal government delivers on its promises.
[The municipality of XX] is honest. Grimmelikhuijsen and Knies, 2017	Q16. The federal government is honest.
The government’s work is open and transparent. World Values Survey, 2017–2021	Q17. The federal government’s work is open and transparent

### Validation of the Trust in Government measure (TGM)

#### Methods for validation study

##### Study sample

Leger was commissioned to administer an online survey to Canadians in French or English from June 1 to June 14, 2022, for the purpose of psychometric analysis of our measure. Participants were eligible to participate in the study if they were Canadian residents and were 18 years of age or older. Individuals self-identifying as FMNI, Black Canadian, low-income (household income <$40,000), LGBT2SQ + and newcomers to Canada (immigrated to Canada $$$$\le$$$$5 years ago) were oversampled to yield a diverse sample including individuals from historically disadvantaged populations. Leger administered the survey using their online platform. A total of 878 individuals completed the survey.

### Approach to criterion validity

We evaluated criterion validity using the following 3 constructs identified in the literature to be associated with trust in government: [1] vaccine hesitancy [8, 10, 11], [2] trust in public health messages and health advice from the government [9], and [3] beliefs in misinformation/conspiracy theories [16]. To examine criterion validity, five variables were derived from survey responses to measure the 3 previously mentioned constructs: [1] vaccine hesitancy, [2] beliefs in vaccine conspiracies, [3] beliefs in COVID-19 conspiracies, [4] trust in public health messages about COVID-19 from the government, and [5] trust in public health advice about COVID-19 from the government.

A score for vaccine hesitancy was measured using the 10-item Adult Vaccine Hesitancy Scale (aVHS) [49] as evaluated on a 5-Point Likert scale (ranging from 1 = strongly disagree to 5 = strongly agree), where higher scores were indicative of higher vaccine hesitancy. Scores on these 10 questions were summed to create an overall score of vaccine hesitancy.

Belief in vaccine conspiracies was measured using the 7-item Vaccine Conspiracy Belief Scale [50] with responses evaluated on a 5-Point Likert scale (ranging from 1 = strongly disagree to 5 = strongly agree), with higher scores indicating higher beliefs in vaccine conspiracies. Scores on these 7 items were summed to create a total score for beliefs in vaccine conspiracies.

Belief in COVID-19 conspiracies was measured using the 6-item COVID-19 conspiracy beliefs scale [51, 52]. Scores were evaluated on a 5-Point Likert scale (ranging from 1 = strongly disagree to 5 = strongly agree), with higher scores indicating higher beliefs in COVID-19 conspiracies. Scores on these 6 questions were summed to create a total score for beliefs in COVID-19 conspiracies.

Two questions on trust in public health messaging and advice (‘The federal government provides trustworthy messages about COVID-19 to the public’ and ‘The federal government provides trustworthy health advice about COVID-19 to the public’) were included on the survey based on past research [53] and responses to these questions were used as outcome variables in two models of the criterion validity analyses. These questions were evaluated on a 5-Point Likert scale (ranging from 1 = strongly disagree to 5 = strongly agree), with higher scores indicative of higher trust in public health messaging and advice. These categories were collapsed into the following: 1 = strongly disagree/disagree, 2 = neither agree nor disagree, and 3 = agree/strongly agree to ensure adequate cell counts for each level of the ordinal variable and for increased ease of interpretation of the logistic regression models described below. Scores were not extremely skewed; therefore, Pearson’s correlation coefficient was used to examine the bivariate associations between survey items. Before conducting further analyses, the correlation matrix and the communality/uniqueness values were examined to identify any potential redundancy for all items.

### Statistical analysis

Descriptive analyses, followed by exploratory and confirmatory factor analyses, were conducted for the purpose of validating the instrument. Descriptive analyses provided the mean, median, standard deviation, skew, and kurtosis of each survey item and were retrieved using the PROC UNIVARIATE function in SAS. The Pearson’s Correlation matrix for all survey items and the Cronbach’s alpha were examined using the PROC CORR function in SAS. The Pearson’s Correlation Coefficients were then imported into Excel where the inter-item correlation (IIC) for the survey items was calculated.

Exploratory Factor Analysis (EFA) was conducted on the survey data using PROC FACTOR in SAS. To determine the number of factors to extract for model testing, eigenvalues (> 1) and the scree plot were consulted. Once the number of factors to extract was determined, several EFA models were built using PROC FACTOR and factor loadings were examined (> 0.40) to assess the merit of the models.

Confirmatory Factor Analysis (CFA) was conducted via PROC CALIS in SAS on the survey data to confirm the factor structures identified in the EFA models. Model fit was evaluated using recommended cut-off criteria for several fit indices including the Comparative Fit Index (CFI) (> 0.90), Standardized Root Mean Squared Residual (SRMR) (< 0.08) [54], and Root Mean Square Error of Approximation (RMSEA) (< 0.10) [55]. Given that the sample size was greater than 200, small deviations in the expected and observed covariance matrices may cause the null hypothesis to be rejected [56], and so a significant chi-square value was not considered a strong indication of model misspecification.

### Validity tests

To evaluate criterion validity for the three continuous variables (vaccine hesitancy, beliefs in vaccine conspiracies, and beliefs in COVID-19 conspiracies), linear regression models were built using PROC LOGISTIC in SAS. To evaluate criterion validity for the two ordinal variables (trust in public health messaging about COVID-19 from the government and trust in health advice about COVID-19 from the government), logistic proportional odds regression models were fitted using PROC LOGISTIC in SAS. The linear and logistic regression models each had a global score of trust in government as the only predictor variable, which was derived by summing scores on responses for each participant to the survey items that were included in the final measure. We expected the following regarding the criterion validity:

1. Trust in government would be negatively associated with vaccine hesitancy;
2. Trust in government would be negatively associated with beliefs in vaccine conspiracies;
3. Trust in government would be negatively associated with beliefs in COVID-19 conspiracies;
4. Trust in government would be positively associated with trust in public health messaging about COVID-19 from the government; and.
5. Trust in government would be positively associated with trust in health advice about COVID-19 from the government.

### Results of validation study

#### Item descriptive statistics

The means of survey items ranged from 2.47 to 3.80 out of 5 with standard deviations (SD) of 1.09 to 1.22. The median of survey items ranged from 2 to 4 and most items had a median score of 3, showing that most questions scored toward the middle of the distribution. The skew of survey items ranged from − 0.95 to 0.51 (M= -0.20), with items ranging from moderately skewed to fairly symmetrical. The kurtosis of items ranged from − 0.90 to 0.42 (M= -0.70), which also suggest that scores are somewhat symmetrical.

The correlation coefficients between items ranged from − 0.03 to 0.84, with an average IIC of 0.56, indicating that the items were moderately correlated. The Cronbach’s alpha for the correlation matrix was 0.96, suggesting a high internal consistency between items. Question 13 [*The federal government puts their political agenda ahead of the wellbeing of the population*] had a weak, negative, and statistically insignificant correlation with most items. Additionally, it had a high uniqueness value (0.99), indicating that it is not related to the underlying latent structure, and so it was removed prior to subsequent analyses. The following two questions were highly correlated (r = 0.82, p < 0.0001): Question 7 [*The federal government carries out its duties very well*] and Question 8 [*The federal government acts in the best interest of citizens*]. Question 7 [*carries out duties well]* was deemed to be captured by other questions assessing competence and was therefore removed to reduce redundancy with Question 8. Additionally, the following two questions were also highly correlated (r = 0.84, p < 0.0001): Question 16 [*The federal government is honest*] and Question 17 [*The federal government’s work is open and transparent*]. The dimension of *honesty* in Question 16 is captured by other questions assessing integrity (e.g., Q14. [*The federal government is truthful in communication with citizens*], Q15. [*The federal government delivers on its promises*], Q17. [*The federal government’s work is open and transparent*] and was therefore removed to reduce redundancy. Question 7 and Question 16 also had high communality values of 0.8 and 0.82 respectively, further supporting the removal of these items.

After removing Questions 7, 13, and 16, the correlations between survey items ranged from 0.24 to 0.79. and the IIC was 0.61, which suggests that the items were moderately-to-strongly correlated. The Cronbach’s alpha for the correlation matrix was 0.96, suggesting a high internal consistency between items.

### Sampling adequacy

After excluding observations that were missing values on any of the survey items, the total sample size was 823, which exceeds the minimum recommendation of 300 subjects for EFA [57]. The Kaiser-Meyer-Olkin (KMO) [58] and Bartlett’s Test of Sphericity [59] statistics were also calculated and used to examine the acceptability of the sample size for EFA. The KMO measure of sampling adequacy returned a value of 0.96 for the correlation matrix, which is interpreted as excellent [60]. The Bartlett’s Test of Sphericity ${\chi }^{2}($91) = 10496.88 p < 0.0001, demonstrating that the dataset is appropriate for factor analysis.

### Exploratory factor analysis

**Fig. 1:**
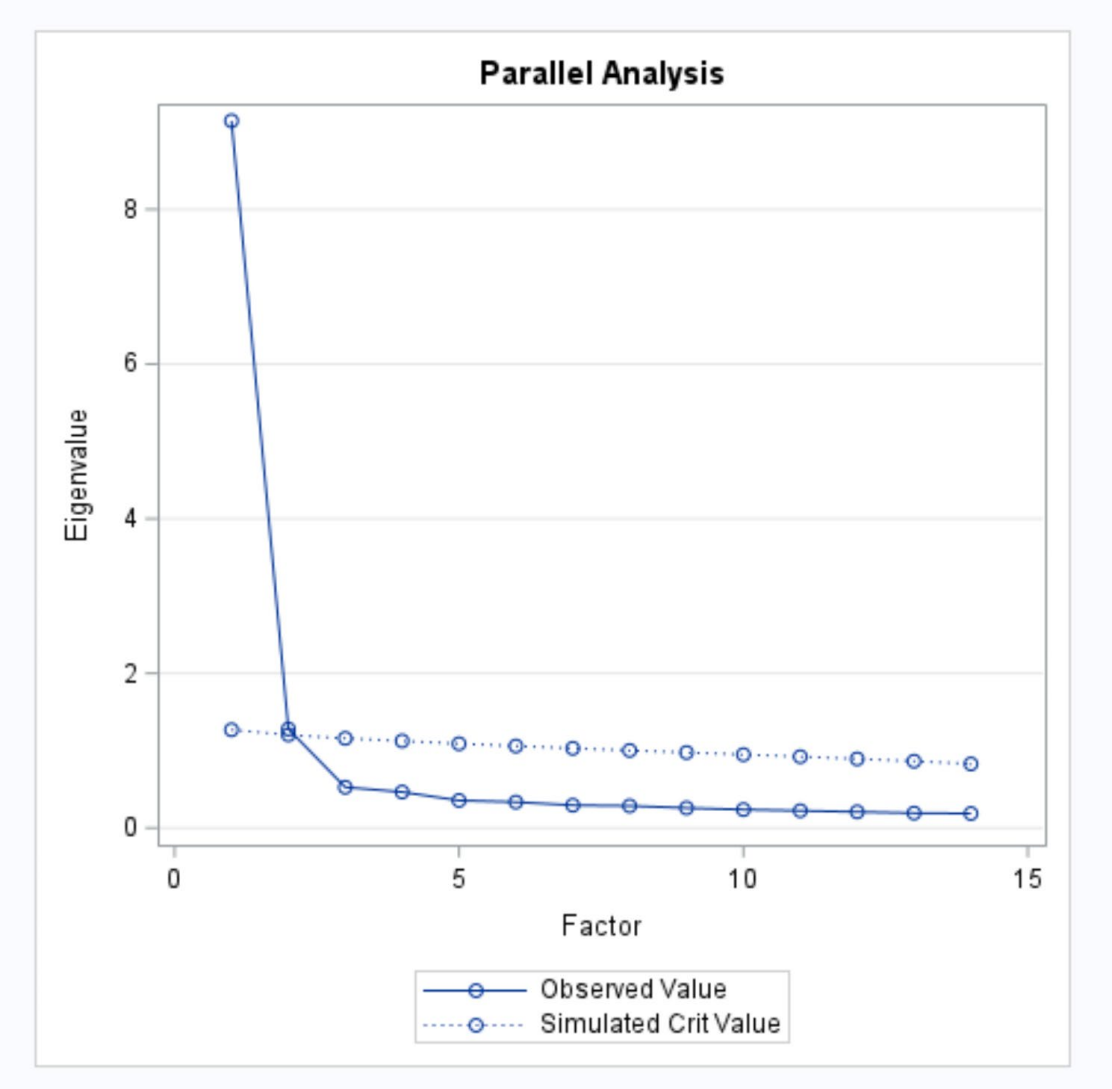
Scree Plot for 14 Trust in Government Survey Questions

Initial Extraction and Number of Factors to Retain. The dataset deviated from multivariate normality based on the Shapiro-Wilk normality test (*W* = 0.95, p < 0.001), therefore the principal factor analysis estimation was used in the initial factor extraction, as it is more appropriate for data that deviate from multivariate normality [61]. The parallel analysis scree plot (Fig. 1) suggests that one factor accounts for a large proportion of the variance, with up to one additional factor. The Eigenvalues for the top three factors were 9.14, 1.28 and 0.52 respectively. Based on the Eigenvalues (> 1) and the scree plot, up to two factors were extracted for the EFA models.

### EFA models

In a unidimensional model with the 14 survey questions, all factor loadings were above 0.40 as shown in Table 3, except for question 1 (factor loading 0.39), suggesting that a unidimensional model is a good fit for the data. Results of the oblique two-factor model conducted using Promax rotation suggested that all questions load onto one factor (Factor 1), except for questions 1 and 2, which formed a separate factor (Factor 2). Unrotated and orthogonal two-factor models were also explored, with similar findings as the oblique rotation model (results available upon request). Given that each factor must have a minimum of three items, a second factor was not evaluated in subsequent confirmatory factor analysis.

**Table 3:** Unidimensional EFA Model with all 14 Survey Questions

Survey Question	Factor Loading
Q1	0.39
Q2	0.55
Q3	0.79
Q4	0.84
Q5	0.88
Q6	0.82
Q8	0.89
Q9	0.83
Q10	0.84
Q11	0.83
Q12	0.86
Q14	0.85
Q15	0.81
Q17	0.82

### Confirmatory factor analysis

Based on the results of the EFA, the items were used to construct the following two models: [1] a unidimensional factor model with all 14 items, and [2] a unidimensional model with question 1 removed. The models used the Least Squares Maximum Likelihood estimation method, which performs better than Maximum Likelihood for data that violate normality assumptions [62].

All items in Model 1 had factor loadings above 0.40 except for question 1, and all items in Model 2 had factor loadings above 0.40. The fit indices for both models meet the recommended cut-off criteria except for the RMSEA in Model 1, which had a score of 0.11 [95% CI (0.103–0.117)]. By comparing the fit indices for Models 1 and 2 (presented in Table 4), it is apparent that Model 2 is a better representation of the underlying data than Model 1, supporting the removal of question 1 and the use of a 13-item unidimensional model. The removal of this question is supported conceptually as well, as this question is focused on the dimension of *capability*, which is also measured in question 2.

**Table 4:** Model **fit Statistics for the Two Unidimensional CFA models**

Model	df	${\chi }^{2}$, p-value	CFI	RMSEA (95% CI)	SRMR
(1) Unidimensional with Question 1	77	848.13, p < 0.0001	0.93	0.11 (0.103–0.117)	0.05
(2) Unidimensional without Question 1	65	456.97, p < 0.0001	0.96	0.09 (0.078–0.093)	0.03

### Criterion validity

Our analyses demonstrate criterion validity as we accept all proposed predictions. Based on the three linear regression models, scores on trust in the federal government were negatively and significantly associated with vaccine hesitancy ($\stackrel{\prime }{\beta }=-0.17,p<0.0001)$, scores on vaccine conspiracy beliefs ($\stackrel{\prime }{\beta }=-0.19,p<0.0001)$, and scores on COVID-19 conspiracy beliefs ($\stackrel{\prime }{\beta }=-0.16,p<0.0001),$ as outlined in Table 5.

**Table 5:** Results of Simple Linear Regression Models with Trust in Government as Predictor Variable

Outcome Variable	Parameter Estimate	Standard Error	t-value	p-value
Vaccine Hesitancy	-0.17	0.01	-12.93	< 0.0001
Vaccine Conspiracy Beliefs	-0.19	0.02	-9.47	< 0.0001
COVID-19 Conspiracy Beliefs	-0.16	0.01	-10.59	< 0.0001

As noted, to examine the association between scores on trust in the federal government and [1] trust in public health messaging about COVID-19 from the government, and [2] trust in public health advice about COVID-19 from the government, proportional odds models were fitted. For ease of interpreting the findings, the levels of the ordinal variables were reversed as follows: 1 = agree/strongly agree, 2 = neither agree nor disagree, and 3 = strongly disagree/disagree. There was a positive association between scores on trust in the federal government and: [1] trust in public health messaging about COVID-19 (OR = 1.16, 95% CI: 1.14–1.18, p < 0.0001) and [2] trust in public health advice about COVID-19 (OR = 1.17, 95% CI: 1.15–1.19, p < 0.0001). More specifically, these odds ratios can be interpreted as:

1. For every one unit increase in scores on trust in federal government, the odds of scoring in the strongly agree/agree category are 1.16 times greater compared to the neither agree nor disagree and the strongly disagree/disagree categories.
2. For every one unit increase in scores on trust in federal government, the odds of scoring in the strongly agree/agree category are 1.17 times greater compared to the neither agree nor disagree and the strongly disagree/disagree categories.

Given these findings, there is sufficient evidence supporting criterion validity of the measure.

### TGM

Based on the review of existing measures, analysis of 56 qualitative interviews, factor analyses and validation testing, we present a 13-item unidimensional scale measuring trust in the Canadian Federal government. The final survey and instructions for sure are available to readers upon request of the corresponding author.

## Discussion

Our aim was to develop and validate a measure of trust in government so that it may be used by government and researchers to measure dimensions of trust over time and inform the careful designing and communicating of public health initiatives in ways which build trust across multiple and diverse communities. This unidimensional scale demonstrated strong internal consistency and test validity, suggesting that trust in government can be measured as a single underlying construct. The unidimensional model also demonstrated strong criterion validity, as measured by the association of scores with vaccine hesitancy, vaccine conspiracy beliefs, COVID-19 conspiracy beliefs, trust in public health messaging about COVID-19, and trust in public health advice about COVID-19.

The finding that all items loaded onto a single factor, leading to a unidimensional model, differs from previous measures whereby trust in government was argued to be best captured by three dimensions [38], or where trust in public health authorities was represented by two dimensions [43]. Our model, in contrast, aligns with Belgian research data from 2002, 2004, 2006, 2008, and 2010, which suggests that trust in political institutions is a one-dimensional latent construct [63]. It may be that the dimensions of competence, beneficence, and integrity are too closely related to be identified as unique factors when measuring trust in government as an institution within the Canadian context. For example, it is unlikely that an individual would find a governmental institution to have integrity unless it also demonstrated competence and beneficence, or that an individual would view the government as competent if it did not also demonstrate beneficence. An alternative explanation for the unidimensionality relates to whether individuals consider organizations as being capable of showing care or integrity. Taylor suggests that people frequently anthropomorphize organizations, describing them as being caring or having integrity [64]. However, it is possible that these qualities can not be ascribed at an organizational level; only to individuals representing the organizations. As such, questions regarding beneficence and integrity are closer to assessments of the competence of an abstract system.

This measure can be used within the Canadian context, or within other high-income countries; for example, in OECD countries already in support of measuring trust (though currently measuring trust in government using a single item [31]) for the purpose of cross-country comparison as it relates to political practices and health. However, given that other measures argue for multidimensionality, researchers might consider tailoring and validating the measure, as well as adjusting the language in terms of type of government (e.g., federal, state, local) for which they are intended [38, 45, 65]. In terms of jurisdiction, it is also important to note that our focus here is Federal, rather than Provincial/State or municipal levels of government. While within Canada the powers of parliament (Federal) differ from those of Provincial government (see [66] the questions used are not specific to the powers held by individual governments (e.g., Federal oversees criminal law and no survey questions are specific to this matter). As such, we support the adaptation of the current instrument for validation and subsequent use in measuring trust in provincial/state government within Canada and other high-income nations.

### Future research

While trust in government has declined at a population level globally, within Canada and elsewhere, there are notable differences in levels of trust between specific equity-deserving sub-groups. While we did oversample historically disadvantaged populations, we did not compare the factor structure of the reported measure across various sub-groups in Canada and recommend this as a next step in future research. While past research has found the factor structure for governmental trust to be consistent across sub-groups [38, 67], future research should examine if this specific measure is consistent for diverse sub-groups (e.g., low- vs. high-income and individuals of diverse sexual identities and orientations). Relatedly, participants included in this study were required to have email access to be recruited and a device with Internet access to complete the survey, limiting the individuals who were eligible to participate in the study. Future researchers may consider examining the validity and factor structure of this measure in other disadvantaged groups that may not have been reached by our recruitment approach. We also note that due to the use of third-party data, we were unable to report a response rate for both the development and validation studies. However, our ability to sample equity-deserving groups by collecting these data through Leger is a strength and outweighs this limitation.

We also recommend that this measure, once validated for subpopulations, be used to measure trust over time in equity deserving groups. For example, within Canada, data from the 2008 General Social Survey reports that trust varies among Canadians, and particularly among populations identified as equity-deserving – that is, populations that have not been advantaged by social institutions to the same extent as more privileged populations. For example, lower trust in government among Indigenous Peoples - rooted in ‘the history of colonialism and betrayal’ by earlier and contemporary governments [68] – is important to measure to determine if Reconciliation efforts help to foster greater trust among these groups. Trust in government may also be lower in other disadvantaged groups such as 2SLGBTQ + and Black Canadians due to historic discrimination [69, 70], low-income Canadians due to the high levels of inequality in the population [71], and young adults [72] which may be related to their increased awareness of global challenges (e.g., financial crises, COVID-19 pandemic, climate change) and general dissatisfaction with governmental approaches to these challenges [73]. As global governments work to redress issues of concern within their population (e.g., redressing inequities, invoking climate change policies), measures of trust might provide a barometer for public support. To (re)build trust across these populations, it is critical that we measure trust over time to inform and evaluate policy interventions. It will, however, be important in the collection and analysis of data to recognize that an individual’s social positions (including but not limited to race, ethnicity, gender, sex, sexuality, class, religion, (dis)ability, and neurodiversity) interact, intersect, and compound to manifest one’s unique lived experience [74], and consequently, their trust. As such, applying a lens of intersectionality in the design and analysis of research is recommended.

We also recommend that researchers consider developing a measure that includes trust at both system and interpersonal levels in one instrument. As noted in our conceptual framework, trust in individuals (e.g., political leaders), who serve as the face of institutions, can affect trust in the organization which they represent, though the causal relationship between these two levels of trust is complex to evaluate. We would expect that questions about government, as an institution, would prompt consideration of the specific individuals we see in news media and on campaign trails; indeed, trust in government as an institution may be in part shaped by perceptions of the care and competence provided by political leadership [31]. This notion has been described by Calnan and Sanford in their discussion of trust in healthcare [75]. Their data suggest that public views about trust tend to focus on more micro level considerations of the doctor-patient relationship and provider expertise rather than broader concerns with how services are run. In the case of government, this would translate to greater focus on perceptions of leadership rather than the institution. This might be addressed by the development of a single measure that allows researchers to measure trust at both interpersonal and system levels, and to determine which of these two levels most impacts the acceptance of government policy as it relates to health behaviour.

It will also be important to use this measure on a longitudinal basis and consider necessary adaptations over time. While we did not aim to examine citizens’ trust in government based on their response to specific situations (e.g., such as the H1NI [10] and COVID-19 pandemics [15, 76]), data informing this measure were collected during COVID-19. As such, the public were acutely aware of government (in)action and thus our research elicited different perspectives of the government than would have been discussed in interviews pre-pandemic. While in some respects this provided us with valuable insights regarding the construct that might not otherwise be of central concern to Canadians, it is critical that we reconsider and revise measures of trust over time as issues shaping trust continue to evolve.

## Conclusion

This is the first study to develop and validate a measure of Canadians’ trust in the Federal government. This study fills this important gap and can be used to measure citizens’ trust in government over time in an ever-changing political climate and identify populations of Canadians with low trust in government that would benefit from efforts to (re)build trust. Given the timing of our data collection, over a year into the COVID-19 pandemic, it will be important for this measure to be validated post-pandemic and, indeed, as societal shifts continue to shape our relationship with government. Our hope is that this measure is widely shared and used by researchers and policy makers to measure and focus on trust in government as a key indicator of societal and public health. Ultimately, these metrics can be used to build trust in government and subsequently the health of populations, as trust in government is a predictor of many important health outcomes.

## Data Availability

The datasets generated and/or analysed during the current study are not publicly available because ethics approvals preclude the sharing of data. However, data may be available from the corresponding author on reasonable request. Materials (the survey) is available upon request from the corresponding author.

## Abbreviations

OECD: Organisation for Economic Co Operation and Development
NGOs: Non government organizations
TGM: Trust in Government Measure
TiPHA: Trust in Public Health Authorities
FMNI: First Nations, Métis and Inuit
LGBT2SQ+: Lesbian, Gay, Bisexual, Trans, Two-Spirit, Queer
aVHS: 10-item Adult Vaccine Hesitancy Scale
EFA: Exploratory Factor Analysis
CFA: Confirmatory Factor Analysis
SRMR: Standardized Root Mean Squared Residual
RMSEA: Root Mean Square Error of Approximation
IIC: Inter-item correlation
KMO: Kaiser-Meyer-Olkin
